# A qualitative study on individual experiences of chronic hepatitis B patients

**DOI:** 10.1002/nop2.100

**Published:** 2017-10-23

**Authors:** Zahra Taheri Ezbarami, Parkhideh Hassani, Mansoureh Zagheri Tafreshi, Hamid Alavi Majd

**Affiliations:** ^1^ School of Nursing and Midwifery Shahid Beheshti University of Medical Sciences Tehran Iran; ^2^ School of Paramedical Sciences Faculty of Paramedical Sciences Department of Biostatistics Shahid Beheshti University of Medical Sciences Tehran Iran

**Keywords:** chronic illness, experiences, hepatitis B, Iran, nursing, patients, qualitative study

## Abstract

**Aim:**

The aim of this study was to explain the perception of patients with chronic hepatitis B regarding problems in the Iranian society.

**Design:**

Descriptive qualitative research.

**Methods:**

In this qualitative study, 27 patients with chronic hepatitis B in Iran were selected through purposive sampling. The data were collected over 22 months, in 2015–2016, by means of semi‐structured interviews and field notes. The interview transcripts were coded using MAXQDA10 software^®^. To extract categories and themes, the thematic analysis approach was used.

**Results:**

The participants’ age ranged from 25–52 years. Analysis of the data revealed seven themes: insufficient self‐care, misperceptions, stigmatization, psychological consequences, failure, spiritual struggle and post‐traumatic growth.

## 1 INTRODUCTION

Hepatitis B virus (HBV) infection is a life‐threatening viral infection leading to acute or chronic liver disease (World Health Organization, 2015a). Worldwide, infections with viral hepatitis B and C are the main causes of hepatocellular carcinoma (Giang, Selinger, & Lee, 2012). Chronic liver disease and cirrhosis are considered as the 12th leading cause of death in the USA and they cause one out of every 10 deaths in the world (Murphy, Xu, & Kochanek, 2013; WHO 2014). According to a systematic review, 248 million individuals were infected by HBV throughout the world in 2010 (Schweitzer, Horn, Mikolajczyk, Krause, & Ott, 2015). HBV and its complications cause over 780 thousand deaths annually (Lozano et al., 2013). Recently, Iran has been classified as an intermediate HBV zone (Mohammadi et al., 2016). The prevalence of hepatitis B in the general population is 2.2% (Vaziri Salehi, Sadeghi, Hashiani, Fesharaki, & Alavian, 2016). Interfamilial contact was one of the major routes of HBV transmission in Iranians (Alavian et al., 2012; Salehi et al., 2012). Although the global vaccination program against hepatitis B had decreased the rate of HBV carriers in Iran, the number of HBV infected cases remains noticeably high (Keyvani et al., 2014).

## 2 BACKGROUND

The transmission of HBV takes place through the parenteral route, sexual intercourse, personal contact and vertical transmission or contact with infected blood. This virus can survive outside the body for at least 7 days and transmission can occur during this period (WHO, 2015b). The likelihood of chronic infection through vertical transfer (mother to child) is about 70–90% (WHO, 2015a).

When an individual becomes infected, the virus proliferates in the liver and is passed through the bloodstream. The long‐term release of HBV into the bloodstream causes individuals to become highly infectious (Yildiz et al., 2015). A persistent infection may bring about the development of cirrhosis and hepatocellular carcinoma (HCC). Furthermore, individual factors including gender, age and family history; and viral factors including HBV DNA, genotypes and hepatitis B surface antigen level; are associated with the development of these complications (Yuen et al., 2016).

Half a century since the discovery of HBV, despite recent significant improvements in treatment regimens, eradication of the virus is not yet feasible (Suk‐Fong Lok, 2016). Moreover, recent studies indicate that almost 40% of those infected with HBV receive no treatment despite the discovery of antiviral treatment (Papatheodoridis, Tsochatzis, Hardtke, & Wedemeyer, 2014). Besides, there is a lack of disease management, control and treatment for those infected with HBV (Yang, 2013). Low health‐related quality of life, lack of knowledge about the disease and psychological problems such as fear, depression and anxiety have been observed in those infected with HBV (Alizadeh, Ranjbar, & Yadollahzadeh, 2008; Levy et al., 2008). In addition, the fear of stigma in these patients leads to a lack of adherence to the treatment plan (Wu, Yim, Chan, Ho, & Heathcote, 2009).

The susceptibility to liver cancer or the possibility of the disease developing into the active phase is a major concern for patients infected with HBV. Life with a chronic illness is a confidential, solitary and unparalleled experience.

Few studies consider the sufferings of patients with chronic hepatitis B specifically. Moreover, these studies have chosen the participants having either hepatitis B or hepatitis C (Hassanpourdehkordi, Mohammadi, & Nikbakhatnasrabadi, 2016; Mohammadi, Hassanpourdehkordi, & Nikbakhatnasrabadi, 2013). But the present study has focused on the patients with hepatitis B who live with their family. Infection of chronic hepatitis B is a confidential and unparalleled live experience for the majority of patients. Researchers conducted by means of qualitative approach adhering to a naturalistic view are free from prejudices and restrictions view existing in positivistic view in quantitative approach. This can be helpful for better understanding of living with chronic Hepatitis B. Qualitative studies previously conducted in Iran have focused on certain aspects of the disease such as self‐care, stigma and psychology problems. (Hassanpourdehkordi et al., 2016; Mohammadi et al., 2013; Valizadeh et al., 2016). Chronic hepatitis is a disease influencing physical health, psychological status and social and spiritual aspects of each individual. Therefore, it is necessary to conduct more studies to expand the knowledge of hepatitis related problems. The qualitative approach was used to capture the unique experiences of patients with chronic hepatitis B during the long span of their disease and to suggest helpful plans which can enhance the quality of life and health of patients.

## 3 METHODS

### 3.1 Design

This descriptive qualitative study, carried out in 2015–2016, used semi‐structured interviews for collecting the data. The study was conducted in the Research Centre of Gastroenterology and Hepatology, which is affiliated to the Guilan University of Medical Sciences, Iran. In addition, health centers affiliated to the Guilan University of Medical Sciences and Guilan Blood Transfusion Organization helped gain access to the desired participants.

### 3.2 Participants

The inclusion criteria were, living with family members; undergoing a confirmatory HBS Ag test; being an inactive carrier of chronic HBV; and absence of other chronic diseases such as diabetes, renal failure, AIDS and hepatitis C. Referring to many studies conducted in Iran, Hepatitis B is mainly transmitted through intra‐familial transmission (Salehi et al., 2012). Based on the results of quantitative studies, infection of hepatitis B is mainly through vertical transmission (mother to child), marital relationships, household contacts with Iranian families (Moghaddasifar, Lankarani, Moosazadeh, Afshari, & Malary, 2016). Regarding these factors, we considered living with family as a main inclusion criteria for participants. As well as different effects of the disease on each individual during different periods of life (adolescence, middle age, motherhood, during pregnancy) and individual reactions caused by different social and cultural components, valuable experiences can be eventuated for an individual playing a distinct familial role. The exclusion criteria were the presence of auditory problems and lack of willingness to participate in the study.

### 3.3 Data collection

Purposive sampling was performed to select patients of different age, gender and educational background, to ensure maximum variation to obtain rich data. Individual semi‐structured interviews were conducted to collect the data. The interviews were commenced with an informal talk to ensure that a dialogue occurred between the researcher and participants and the participants were assured that confidentiality would be maintained.

Subsequently, open‐ended question were asked, such as: “When and how did you realize that you are infected with HBV?” Based on the interviewees’ responses, follow‐up questions were asked to clarify the concept of the study (for instance, “Can you explain more?” or “Can you provide an example?”). Analysis of the data collected from each interview directed the next interview. The sampling continued until the data were saturated. Thus, 27 patients were included in the study. Each interview lasted for 40–120 min, with an average of 57 min. The data were collected over 22 months in 2015–2016.

### 3.4 Data analysis

All interviews were conducted by a researcher and each was transcribed verbatim after recording. Subsequently, the transcripts were read several times while listening to the recorded voices for immersing oneself in the data. Each transcript was imported into MAXQDA 10 software^®^, to organize and categorize the information provided by the participants, to analyze the content of the interviews. Merrill and West's (2009) thematic analysis model was used for the analysis. The transcripts were coded, following which the codes were compared. In the next stage, they were classified into categories and finally, the themes were extracted.

### 3.5 Rigour

To ensure rigour in the study, Guba and Lincoln's (1989) assessment criteria were used. Prolonged engagement with the data, member checks and data triangulation with maximum variation in sampling were ensured. The conformability of the findings was assessed by two external experts who were familiar with qualitative research.

### 3.6 Ethical considerations

The sampling process was conducted after obtaining approval from Local Ethical Committee and a referral from the University to enter the research context. The ethical principles of seeking informed consent from participants and ensuring the confidentiality of their personal information were followed. The place and time of the interviews were determined based on the participants’ agreement.

## 4 FINDINGS

**Figure 1:**
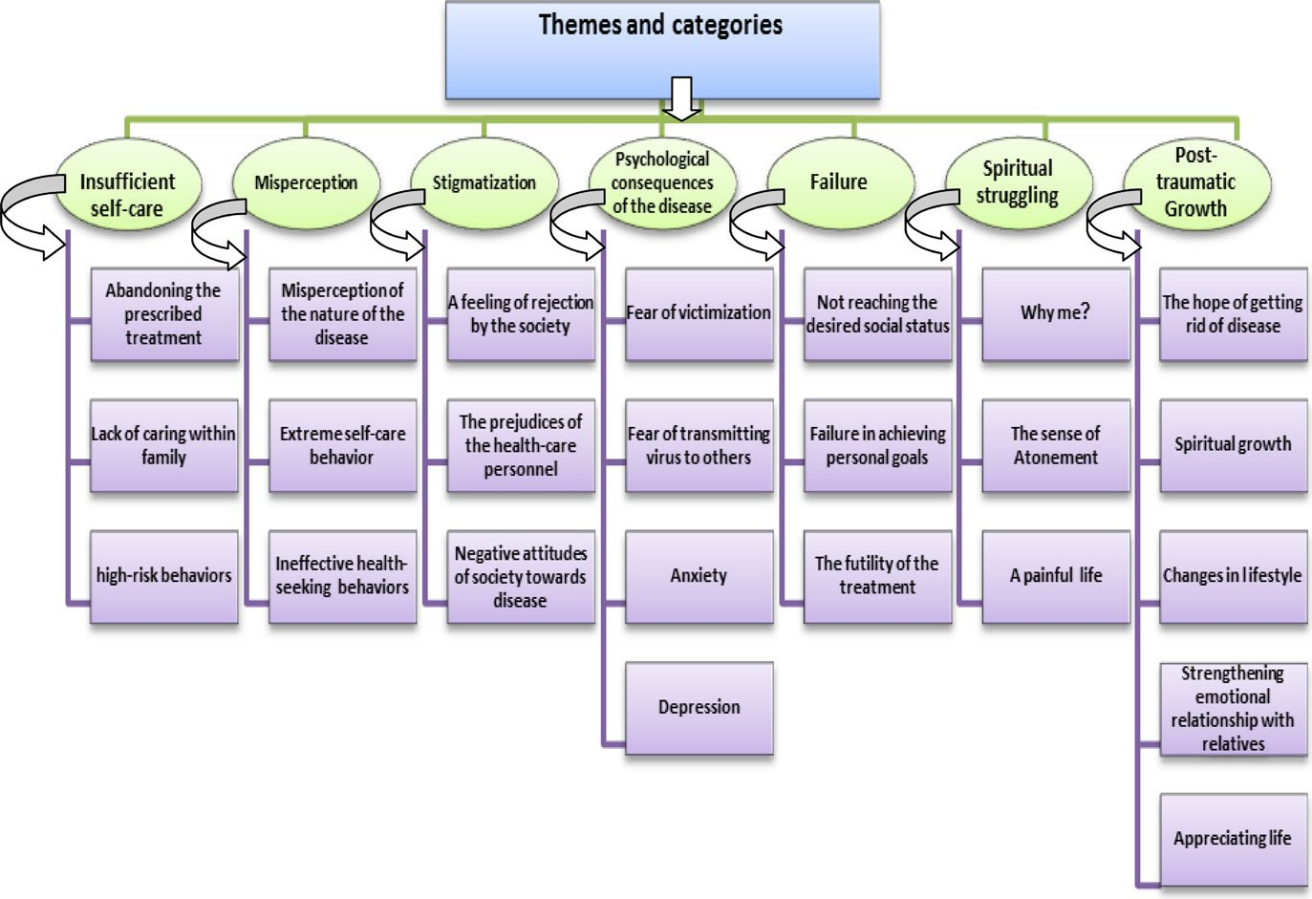
Themes and categories related to individual experiences of patients with chronic HBV infection

The participants of the present study were 27 patients with HBV infection whose duration of disease was 10.5 (SD 8.7). The patients’ ages ranged between 25 and 52 years and their mean age was 36 (SD 8.07). Other demographic characteristics are mentioned in Table 1. The findings of the interviews with the participants, illustrated in terms of the following seven themes, have been summarized in Fig. 1.

**Table 1:** Demographic characteristics of participants (*N *=* *27)

Variable	*N*	%
Gender		
Male	15	55/6
Female	12	44/4
Marriage		
Married	23	85/2
Single	4	14/8
Education		
Did not complete high school	4	14/8
High school diploma	14	51/9
Postsecondary	9	33/3
History of HBVin family		
Yes	13	48/1
No	14	51/9

### 4.1 Insufficient self‐care

Because of the specific nature of chronic hepatitis B, medical care and self‐care principles are essential at different stages of life to prevent liver complications in patients and to control the spread of disease among healthy members of the family. However, the expressions of majority of the participants indicated the opposite. The above theme included three following categories:

#### 4.1.1 Abandoning the prescribed treatment

Most of the participants did not conform to the healthcare schedules recommended by their doctors for several reasons including the lack of obvious physical symptoms and receiving appropriate information, financial problems and suffering from chronic diseases. One of the participants stated:

“My husband and I are infected with the disease in a way that I check my tests every 6 months and my husband every 3 months, but honestly, we do not refer frequently since the costs of the tests are extremely high. Actually, this is a great concern in our family, which prevents any further follow‐up action” (p. 3).

#### 4.1.2 Lack of care within the family

Despite the likelihood of disease transmission in the family through household contact, after being infected with the disease, majority of the participants did not take steps to prevent the transmission of the disease to the family members because of the prejudice or not having disclosed that they had the disease. A patient who had been suffering from the disease for 30 years said, “I take care of myself without informing my family about my disease since they may become anxious” (p. 8).

#### 4.1.3 High‐risk behaviours

It is necessary to change risky behaviours such as alcohol consumption and abstention from high‐fat foods by individuals with fatty liver. However, many participants were found to continue such behaviours. One of the participants said: “My mother is suffering from cirrhosis due to hepatitis and I have to consider the food so carefully. Similarly, my brother, who is also infected with hepatitis, has a drinking habit, which makes his liver condition poorer. The doctor recommended him not eat meat, but unfortunately, he is accustomed to having barbecued food every day” (p. 3).

### 4.2 Misperceptions

The patients also suffered owing to the misperceptions regarding the disease. The following categories were included in this theme:

#### 4.2.1 Misperception regarding the nature of the disease

Lack of familiarity with the nature of hepatitis, its complications and reliance on personal information led to misperceptions regarding the disease. The misperception of the concept of inactive carriers of the disease (known as healthy carriers) was evident from the statements of some of the patients, such a: “I have not been controlling myself since a couple of years. The doctors told me that I am a healthy carrier and should not take any action. Until now, I did not inform my family members that I am infected with hepatitis so that they could be careful. In addition, I have only a vague idea of what a healthy carrier exactly means” (p. 1).

#### 4.2.2 Extreme self‐care behaviour

In other patients, obligation to self‐care was extreme. Owing to their worry about disease transmission, some patients avoided close contact with their children. Some others talked about the extreme precautions that they followed at home, such as, “I'm told that my disease could be transmitted through blood, but I take care of myself at home so that my children and husband could be safe from the disease. Even during cooking, I wear multi‐layered gloves and never cook without gloves” (p. 2).

#### 4.2.3 Ineffective health‐seeking behaviours

Asking for help from unfamiliar sources after the disease has been confirmed, searching for information in the cyberspace and using traditional treatments such as phelebotomy contribute to misperceptions regarding the disease. A highly educated participant said: “I studied about the treatment of the disease regularly by reading magazines or on the Internet. I am very interested in this respect. My sister says the time when I got rid of the illness, I had eaten lots of cherries” (p. 6).

### 4.3 Stigmatization

Another problem faced by patients was related to the cultural and social aspects. In their opinion, the disease leads to stigmatization. This theme included the following three categories:

#### 4.3.1 Feeling of being rejected by the society

According to the statements of several patients, people avoid contact with someone that has the disease. A 25‐year‐old HBV patient said: “People's view about the disease is so unpleasant. In offices, if colleagues know about the disease of an employee, they misbehave with the infected person” (p. 16).

#### 4.3.2 Prejudices of health‐care personnel

Concerns of health personnel related to the possibility of disease transmission and standard precautionary behaviours during the health care had led to bitter experiences for the patients. One of the participants talked about the humiliating behaviour of one of the health‐care personnel during a normal delivery: “when I was giving birth to my child, you don't know how she shouted at me, as if I have committed murder” (p. 3).

Another patient had a bad experience with precautionary actions of hospital staff in dealing with infectious diseases and considered it as a kind of labeling: “After curettage, they put me in an isolated room that had ‘infectious patients’ isolation room’ written on the door” (p. 18).

#### 4.3.3 Negative attitudes of the society towards the disease

Experiences of the patients indicated that people considered hepatitis like AIDS. Once participant reported: “The greatest problem I have with people is that they leave me alone when they are informed about my disease, even at the time of marriage. For example, I had a friend who I told about the result of the HBS Ag test. Not surprisingly, he said, ‘you have HIV,’ and left me forever” (p. 12).

### 4.4 Psychological consequences of the disease

The psychological consequences of the disease caused problems for the patients in the following categories:

#### 4.4.1 Fear of victimization

The feeling of exposure to chronic liver damage and its consequences led to the fear of victimization in patients. A man who had been suffering from the disease for 25 years said: “My father died in this hospital owing to the cirrhosis caused by hepatitis. This disease has created a fear of death in me” (p. 16).

#### 4.4.2 Fear of transmitting the virus to others

The patients were concerned about transmitting the disease to their spouse and children. Therefore, they feared making contact with others. This fear increased over time. As reported by one patient: “In one of the family parties, a child's nail touched a boil on my face. I washed the child's hands with plenty of water, but I was disturbed for several nights that I may have transmitted the disease to this innocent kid. I consulted a health expert till I felt relaxed about it” (p. 6).

#### 4.4.3 Anxiety

Patients’ statements represented distressing thoughts, not pursuing the HBV treatment plan due to adverse psychological effects and feelings anxiety. A young boy who had been suffering from disease for 7 years said: “The disease had a great impact on my mood. Especially, in the first five or six months, I was in a terrible mood and the stress of the disease was disappointing me. Hence, I decided not to follow it up any more and threw out all my test results” (p. 10).

#### 4.4.4 Depression

According to the patients’ reports, depression was another psychological consequence of the disease. One participant said: “The disease affects me mentally. At this time, when I could be a happy young girl and enjoy my life, I suffer from this disease that fills me with the fear of death. It really depresses me” (p. 12).

### 4.5 Failure

Patients’ experiences indicated that being rejected due to the disease in different stages of life led to undesirable feelings that prevented them from accomplishing their goals. The following categories were included in this theme:

#### 4.5.1 Not reaching the desirable social status

The statements of the participants indicated that being rejected from their favorite sport and sport club and lack of access to expected social situations led to feelings of frustration. One participant said: “I've had this problem for 11 years. I played professional football, but because of my disease, I left my favorite sport. I was a good player” (p. 17). He added: “I began working in a large company, but I was fired due to my disease” (p. 17).

#### 4.5.2 Failure in achieving personal goals

Participants reported that their current positive status of HBS Ag acted as an obstacle to achieving a health certificate that is an essential requirement for the recruitment of applicants in Iran. A young girl stated: “My illness prevented me from reaching my goals in life. I'm disappointed. One of the best students of the University was in love with me for more than 3 years. He left me when I informed him about my disease. Besides, despite being a top student myself, I lost several good job opportunities due to the fear of disclosing my disease” (p6).

#### 4.5.3 The futility of treatment

Most participants reported that they participated in treatment projects in the hope of eradicating HBV, but they had not achieved the expected outcome. One of the participants, who did not follow his treatment plan regularly, said: “A few years ago, I was treated by interferon in the hope of being cured of the disease, but it had no effect on me” (p. 20).

### 4.6 Spiritual struggles

The experiences of most participants indicated that they pondered about this problem privately. They thought about why they had been victimized by the disease and what sin they had committed that God created such a destiny for them. The following categories were included in this theme:

#### 4.6.1 Why me?

After being exposed to the disease, the patients often pondered over the questions, “why me?” One of the participants expressed: “I think I'm not a lucky guy. It's true that I'm one of the healthy carriers of the disease, but I don't know why God planned this for me and why I should suffer from this disease” (p. 7).

#### 4.6.2 The sense of atonement

Several patients considered hepatitis as a punishment by God in response to their faulty deeds of the past. One of the male participants said: “Most of the patients are very concerned about how they got the disease and this issue annoys them. I think that all patients assume that they contracted the disease because of their wrongdoings” (p. 5).

#### 4.6.3 A painful life

According to the participants, the disease leads to a painful life, a permanent feeling of annoyance and distressing private thoughts. A participant said: “I feel I shouldn't think about the disease because it annoys me” (p. 1).

### 4.7 Post‐traumatic growth

The participants’ experiences often revealed that despite facing numerous problems, hepatitis could have the following positive psychological implications:

#### 4.7.1 The hope of getting rid of the disease

The expectation of getting rid of the disease was observed in the statements of majority of the participants. A participant said: “Always, from the bottom of my heart, I'm hoping that someone tells me that this disease is definitely curable” (p. 10).

#### 4.7.2 Spiritual growth

The disease causes patients to pay more attention to spiritual beliefs such as relying on religious values and accepting the disease as the will of God. Several patients reported that they considered the disease as the wisdom of God and accepted it. One participant reported: “To cope with the disease, I believe that I should trust in God and that whatever He has given to us is wisdom. This point of view has helped me a lot in dealing with my disease. I think if people rely on spiritual support, most of their problems will be solved” (p. 22).

#### 4.7.3 Changes in lifestyle

After being infected with HBV, several patients tried to change their lifestyle and improve the quality of their life by abandoning high risk behaviours, engaging in effective help‐seeking behaviour and finding ways to become happier. A prolonged carrier of HBV said: “The illness has made my life move very slowly. For example, I lessen my official work, control my weight and stay away from stressful situations. This way, I am able to lead a better life. Otherwise, I could have developed another disease by now” (p. 8).

#### 4.7.4 Strengthening emotional relationships with relatives

Patients’ relatives play a crucial role in accepting the disease and providing the patient with emotional support. A participant said: “My wife cares a lot about me. She does not let the family eat outside food. She has paid attention to my eating habits over these 25 years. She is always in the kitchen!” (p. 16).

#### 4.7.5 Appreciating life

Eventually, some patients could cope with the disease and became more careful about their quality of life. One of the participants said: “Maybe, hepatitis is a better alternative to other terrible events that could occur to me. I could have suffered from a fatal cancer instead. So it is important to live better from now on, despite the problems related to the disease” (p. 3).

## 5 DISCUSSION

Long‐term experiences of patients with chronic hepatitis B were examined in the present study, by using thematic analysis. The participants’ experiences showed that they did not pay sufficient attention to self‐care programs. Studies conducted in European countries indicate that a significant proportion of people suffering from chronic HBV and HCV infections do not receive any treatment at all (Papatheodoridis et al., 2014). Another study showed that the use of antiviral drugs has been safe and effective for up to 5 years after the treatment and that the long‐term suppression of HBV have caused the regression of fibrosis and cirrhosis in patients (Marcellin et al., 2013). Furthermore, results of the present study are compatible with previous research that shows that hepatitis B patients have difficulty in performing self‐care in Iran (Mohammadi et al., 2013).

Most participants in this study exhibited misperceptions about the disease. A similar lack of knowledge about hepatitis has been reported in many studies (Ng, Low, Wong, Sudin, & Mohamed, 2013; Wallace, Mcnally, Richmond, Hajarizadeh, & Pitts, 2011). The feeling of stigmatization revealed in the present study is compatible with the findings of previous qualitative studies showing stigma in patients of HBV infection in Iran (Hassanpourdehkordi et al., 2016; Valizadeh et al., 2016). Patients’ stigmatization‐related perceptions and responses vary across communities, diseases (Butt, 2008) and periods of time. A qualitative study conducted in the British Columbia showed that the main cause of stigma was attributed to misunderstandings regarding the causes and transmission modes of this disease and its association with illicit drug use (Butt, Paterson, & Mcguinness, 2008).

The noticeable psychological consequences of this study were like the results of another study conducted in Iran, which reported that patients with hepatitis B reported feelings of sadness and concerns about death resulting from the disease (Valizadeh et al., 2016). Furthermore, the fear of victimization and transmitting HBV to others reported in present study was compatible with the findings of another study conducted in Iran (Alizadeh et al., 2008). As reported in the present study, experiences of patients with chronic hepatitis C showed disrupted psychological well‐being (Hill, Pfeil, Moore, & Richardson, 2015). Besides, according to a quantitative study conducted in Italy, the relationship between chronic hepatitis C and recurring brief depressions was observed even in the absence of interferon treatment and this relationship was not affected by drug or alcohol dependence (Carta et al., 2012).

In this study, failure to accomplish the self‐ideals and the feeling of futility of treatment led to a sense of frustration over the years. In this context, the results of a study conducted in Pakistan showed that prejudices in the family and the society caused a sense of frustration and isolation in patients with hepatitis B and C patients (Rafique et al., 2014).

Another theme of the present study was spiritual struggles. In the Iranian culture, many people regard each disease as a punishment for sins or atonement for sins. Empirical evidence shows that these struggles lead to poorer health and well‐being (Abu‐Raiya, Pargament, & Exline, 2015).

However, despite all the difficulties recognized by the patients, some exhibited post‐traumatic growth. Similarly, results of a study on HIV‐positive people showed that patients who disclosed their disease to their partners and were supported spiritually exhibited higher post‐traumatic growth (Kamen et al., 2015). Another study on the experiences of HIV patients in Saudi Arabia showed that most of the participants were more religious and frequently used spirituality as a means of coping (Omer, Lovering, & Al Shomrani, 2014).

This qualitative study provided a deeper understanding of the experiences of patients with chronic hepatitis B. Nurses and other health providers have an important role in designing interventions that may convince patients to follow treatment programs and engage in better self‐care. Although providing clinical information alone has been found to improve adherence to treatment, stigma can hold back adherence (Sublette, Smith, George, Mccaffery, & Douglas, 2017). Patients with chronic HBV infection culturally and linguistically need appropriate education, especially at the time of diagnosis. Health providers of patients with HBV infection should improve their knowledge, skills and motivation to ensure better management of their infection (Wallace et al., 2011). A study on patients with hepatitis C revealed that specialist nurses can help patients in regions without proper facilities (Grogan & Timmins, 2010).

In case of chronic disease management, e.g., hepatitis, the spiritual struggles of patients should be controlled with appropriate interventions. Negative spiritual beliefs have been found to have an adverse impact on the physical and mental health of patients, while positive spiritual beliefs are associated with appropriate mental health (Jones et al., 2015).

### 5.1 Study limitations

Patients suffering from hepatitis B were reluctant to reveal their illness and did not visit Medicare centers owing to the stigma. Hence, many of qualified chronic Hepatitis B patients were reluctant to participate in this study, though they were asked to participate in it. The participants in the present study were invited by the researcher gaining their trust by creating a favorable research atmosphere where bilateral relations were based privacy and confidentiality. Interviews were conducted in a quiet room in the Research Centre so as to assure patients about the confidential and private manner of the interviews. Due to the existence of stigma attached to Hepatitis B in the study context, participants may have not spoken freely during the interviews. So, it seemed to be necessary for interviewer to build a calm atmosphere to let participants have a respectful and trustful condition for interview. As this study was qualitative and due to the limited number of participants, the obtained results cannot be generalized to the whole population of chronic hepatitis B carriers.

## 6 CONCLUSION

People suffering from hepatitis B live with their family and interact with people and the society. These people have different healthcare needs throughout various stages of life, such as in adolescence, married life, pregnancy, etc. Hence, it seems necessary to educate patients visiting Medicare centers using effective means throughout the life stages, as well as to provide nursing care without labeling. In turn, this will help patients adjust better to the chronic condition and will improve their quality of life.

To improve patients’ capacity of overcoming the illness and its consequences, nursing education programs should include the comprehensive assessment of hepatitis B problems and the coping strategies employed by patients. Hence, it seems necessary to revise the nursing curriculum to help them work efficiently in regions having a high burden of hepatitis B.

Educational programs help improve the knowledge of nursing related to hepatitis (Frazer, Glacken, Coughlan, Staines, & Daly, 2011); therefore, it seems necessary to upgrade nursing graduates’ knowledge and information about HBV patients’ care needs through retraining programs. Moreover, owing to the impact of cultural aspects on discriminative behaviours and stigma faced by patients, public awareness about the disease can be promoted by the healthcare system, with the help of the media at the community level.

## 7 RELEVANCE TO CLINICAL PRACTICE

Capturing the unique experiences of chronic hepatitis B patients help develop plan to enhance the quality of life of patients. Nursing intervention, such as provision of emotional support, unlabeled care and necessary information to patients can help patients adjust to chronic hepatitis B. Therefore, nursing education programs should include the comprehensive assessment of hepatitis B related problems in the regions with a high burden of hepatitis B. In addition to nurses, other healthcare practitioners, family members and social networks should help patients promote their individual coping capacities and self‐care behaviour.

## CONFLICT OF INTEREST

The authors declare no conflicts of interest in this study.
